# The Perpetual Bath

**Published:** 1884-12

**Authors:** David Prince

**Affiliations:** Jacksonville, Ill.


THE PERPETUAL BATH.

*Messrs. Editors:*—The reading of your editorial in the August number of your valuable journal, reciting what is being done by Hebra and the perpetual bath, induces me to send you some notes of my own use of this agent during several years.

I am free to say that the knowledge of what Hebra had already done, aided me in the conception and in the execution.

Some account of my observations has already been published in the *St. Louis Medical and Surgical Journal*, in 1880 and 1881, after having been presented to the meeting of the Tri-State Medical Society, in Louisville, Nov., 1880.

Dr. Ferdinand Hebra (in his great work, " Diseases of the Skin," Vol. I, p. 320, New Sydenham Society edition,) speaks of having tried in three cases of burns, the perpetual bath at 90° to 100° F.

Accurate observations in his cases, written down from hour to hour, showed no marked change in pulse, respiration or temperature.

I. *The Perpetual Bath by Irrigation.*—N. B., aged 15, lacerated wound of fore arm, from a shot gun, March 12, 1873; entrance on the palmar surface, exit on the dorsal surface, leaving the olecranon process untouched, but displaying 7.5 centimeters (three inches) of the length of the ulna and lacerating the soft parts the length of 10 centimetres (4 inches). The anterior opening, powder burnt, was 19 millimetres (three-fourth inch) in diameter.

The wound was managed by placing it upon a splint slightly flexed, and applying every thirty minutes a solution of carbolic acid of the strength of about one per cent. There was not any redness along the lacerated margins of the wound at any time.

On the 27th day, slight hemorrhage occurred, and in the evening several spoonfuls. The bleeding artery was tied, and on exploring the wound a sharp pointed fragment of bone was found, which is supposed to have ulcerated in the ulnar artery. There never was any redness along the cutaneous margins during the whole progress of healing.

II. *Perpetual Immersion.*—W. B., aged 14. Forearm lacerated by a shot gun, the charge spending its force in comminuting the radius and the ulna, flattening the soft parts as the extensive laceration spread open (Nov. 24, 1879).

The forearm was kept in a small bath during 122 days, at a temperature of 37°–39° C. (99° to 101° F.), maintained by an alcohol lamp, the flame of which was graduated to the effect desired. The water of the bath was medicated with carbolic acid, one part in one thousand, together with salicylic acid, one part in ten thousand.

On the 60th day, a small abscess was lanced under water. On the 63d day, the first muscular slough was detached—the muscular fibers beautifully macerated, and infiltrated with pus corpuscles and crystals of salicylic acid. On the 71st day, a small spiculum of bone, 37 millimetres long and 16 m. thick, was pulled away.

This piece of bone was entirely free from odor.

On the 112th day, there was the first detachment of dead skin, the adjoining living skin having at no time exhibited any redness.

On the 122d day, the limb was taken from the bath and the patient was permitted to sit up.

On the 143d day, a portion of the wad introduced by the discharge came away from near the elbow joint.

On the 183d day, a fragment of the ulna came away, about 10 m. in all its diameters.

The case recovered, with motion and sensation in all the fingers, to a limited extent. The tactile sensibility, at first entirely lost in the little finger, became partially restored.

*Sixty-five Days in a Bath Tub.*—W. N., aged 17, April 26, 1878, had been fourteen weeks under treatment on account of inflammation, involving extensive necrosis of both femur and tibia.

Very large bed sores had been developed, on account of which the patient was rested upon a canvas, stretched across a frame, and let down into a bath.

He liked this so well that it became his continuous bed. He ate, drank, slept and evacuated in the bath, and screamed if an attempt was made to take him from the water. The temperature was maintained by the inflow of warm water, an equal outflow going on at the same time. Once a day the water was completely drawn out and the tub cleaned.

On the 19th day, the bed sores had become nearly healed.

Toward the last, his forces failed, and he finally died July 18th, having been sixty-five days in the bath.

This case is valuable as showing the friendliness of immersion to the progress of granulation and cicatrization.

The case furnishes encouragement for the employment of immersion in cases of severe lacerations, such as occur in railroad accidents.

With the employment of chloride of sodium, carbolic acid and salicylic acid in sufficiently weak solution, the fear of blood poisoning from the absorption of fluids in the incipient period of decomposition may be laid aside, and the disappearance of shock averted, for such amputations as may be necessary. It is at the same time a question whether or no this is the best management for shock.

*Extrophy of the Bladder—The Employment of the Bath after the Operations.*—Wm. St. John, of stout build and good general health, an opium eater, taking an ounce of laudanum at a time and ten ounces every day. Extrophy of the bladder and shortening of the penis with epispadias. Absence of the symphysis pubis and the pelvis spread, making the transverse diameter from the extended surface of one trochanter to that of the other 625 of a meter (26½ inches). The operation, Dec. 8th, 1874, was made according to the third method of John Wood, exposing a very large amount of raw surface. As the only purpose of this paper is to relate observations upon the use of the bath, it is not necessary to enter into details. (The case was published in the St. Louis *Medical and Surgical Journal*, November, 1881).

The first dressing of the wound was done on the 2nd day, in a large sitz-bath, and repeated each day in the bath; the patient being allowed to remain as long as he pleased, the temperature being regulated according to the sense of comfort.

The laudanum taken during the period varied from 120 to 180 cubic centimeters (4 to 6 ounces) a day. On the 10th day the patient was well enough to sing. On the 26th day the patient stood for a photograph. On the 42nd day an additional operation was made. The next day after this operation, the pulse was 116 and the temperature 38°,3+C. (101° F.), and at the end of thirty-six hours the patient had been thirty-two and a half hours in the bath.

The following table shows the time spent in the water on the days succeeding this; the comfort of the patient being the criterion of fitness:

| | | | |
|---|---|---|---|
| 3d day | 18 hours | 8th day | 22 hours. |
| 4th " | 14 " | 9th " | 16 " |
| 5th " | 14 " | 10th " | 15 " |
| 6th " | 14 " | 11th " | 16 " |
| 7th " | 18 " | 12th " | 15 " |

After this, the average time in the water was twelve hours, or from 9 A. M. to 7 P. M. When out of the water the wound was subject to a perpetual irrigation with weak carbolized water.

July 24th, or on the 75th day from the first, a third operation was made, and on the 27th, or the third day from the operation, he had been fifty hours in the bath.

March 22nd, or the 104th day, the fourth operation. This patient ultimately made a very satisfactory triumph of surgery.

It was found impossible to diminish the consumption of opium during the surgical treatment.

During a great portion of the treatment he took daily 4 c.c., (a drachm) of the tincture of chloride of iron combined with

66 c.c. (10 minims) of nitric acid. Quinine was given in various quantities during the whole period.

This case certainly indicates the employment of the bath after operations and injuries exposing a considerable amount of raw surface.

Another important occasion for the bath is the condition following lithotomy and other perineal sections. If the bath is employed as often and as long as the exudations and the flowing urine produce any irritation, there is pretty certainly a washing away of all material liable to go into septic decomposition.

The relief by opium is deceptive, permitting septic poison to enter without notice; but the relief by diluting and floating away decomposing material, adds to the element of comfort, that of safety.

One of the conditions most frequently demanding the treatment so successfully practiced in the case of St. John (just related) is that of puerperal patients suffering septic poisoning from decomposing material in contact with lacerations or within the interior of the uterus, which is in the physiological condition of a laceration, open to septic ingress.

In all private houses it is practicable to improvise a sitz-bath for the pelvic bath, or a full length bath for wounds and diseases of other parts of the body; and in all public institutions it is a wonder that there is not oftener the welcome sight of a bed and a bath-tub along side of each other.

If the patient is very helpless he can be easily lifted from the bed to the bath, and when sufficiently soothed by the contact of warm water, and the temperature brought to the degree of comfort, he can be lifted out again to receive the alternation according to the dictate of comfort.

Respectfully,

Jacksonville, Ill. DAVID PRINCE.

